# A Video Game Intervention to Prevent Opioid Misuse Among Older Adolescents: Development and Preimplementation Study

**DOI:** 10.2196/46912

**Published:** 2023-11-03

**Authors:** Kammarauche Aneni, Claudia-Santi F Fernandes, Lily A Hoerner, Claire Szapary, Tyra M Pendergrass Boomer, Lynn E Fiellin

**Affiliations:** 1 Child Study Center Yale University School of Medicine New Haven, CT United States; 2 Biomedical Informatics and Data Science Yale University School of Medicine New Haven, CT United States; 3 Department of Internal Medicine Yale University School of Medicine New Haven, CT United States; 4 Yale School of Public Health New Haven, CT United States

**Keywords:** videogames, serious games, opioid misuse, mental health, adolescents

## Abstract

**Background:**

Opioid misuse and mental disorders are highly comorbid conditions. The ongoing substance misuse and mental health crises among adolescents in the United States underscores the importance of widely scalable substance misuse preventive interventions that also address mental health risks. Serious video games offer an engaging, widely scalable method for delivering and implementing preventive interventions. However, there are no video game interventions that focus on preventing opioid misuse among older adolescents, and there are limited existing video game interventions that address mental health.

**Objective:**

This study aims to develop and conduct a formative evaluation of a video game intervention to prevent opioid misuse and promote mental health among adolescents aged 16-19 years (*PlaySmart*). We conducted formative work in preparation for a subsequent randomized controlled trial.

**Methods:**

We conducted development and formative evaluation of *PlaySmart* in 3 phases (development, playtesting, and preimplementation) through individual interviews and focus groups with multiple stakeholders (adolescents: n=103; school-based health care providers: n=51; and addiction treatment providers: n=6). *PlaySmart* content development was informed by the health belief model, the theory of planned behavior, and social cognitive theory. User-centered design principles informed the approach to development and play testing. The Exploration, Preparation, Implementation, and Sustainability framework informed preimplementation activities. Thematic analysis was used to identify themes from interviews and focus groups that informed *PlaySmart* game content and approaches to future implementation of *PlaySmart*.

**Results:**

We developed a novel video game *PlaySmart* for older adolescents that addresses the risk and protective factors for opioid misuse and mental health. Nine themes emerged from the focus groups that provided information regarding game content. Playtesting revealed areas of the game that required improvement, which were modified for the final game. Preimplementation focus groups identified potential barriers and facilitators for implementing *PlaySmart* in school settings.

**Conclusions:**

*PlaySmart* offers a promising digital intervention to address the current opioid and mental health crises among adolescents in a scalable manner.

## Introduction

### Background

The opioid crisis in the United States has affected many individuals and families. Although opioid misuse among adolescents and young adults has been declining in the past few years, death from opioids among this age group has been on the rise [1]. As a result of the COVID-19 pandemic, the mortality rate from drug overdose among adolescents aged 14-18 years has increased by 94% between 2020 and 2021 [1] and again by 20% between 2021 and 2022. The total number of fatal overdoses for 2022 was 1146 [1]. Opioid-related deaths accounted for up to 85% of drug-related deaths among adolescents between 2020 and 2022 [1].

In 2020 alone, 1.4 million young adults aged 18-25 years and 396,000 adolescents aged 12-17 years misused opioids [2]. Opioid misuse is “the unhealthy use of opioids to alter reality, bring about pleasure or relieve stress” [3]. It includes the use of heroin and prescription opioids in a different way than prescribed by a physician [2,4]. Opioid misuse among adolescents increases the risk of developing an opioid use disorder (OUD) and the risk of dying from opioids [5]. Opioid misuse can also increase the risk of negative mental health outcomes and lead to suicidal thoughts and behaviors [6]. Conversely, preexisting behavioral disorders, such as depression [7], anxiety [8], adverse childhood experiences [9] and posttraumatic stress disorder [10], and misuse of cannabis [11] and alcohol [12] increase the risk of opioid misuse. Approximately 65% of youth with substance use disorder have a co-occurring mental health disorder, and adolescents with these dual diagnoses are at an increased risk of adverse medical outcomes, suicide, incarceration, and interpersonal difficulties at school and at home [13,14]. This dire situation underscores the urgency for targeted preventive interventions that are easily accessible to adolescents and that address the intersection between opioid misuse and mental health.

Opioid misuse typically starts during mid- to late adolescence [15], suggesting that this is a crucial period for targeted and accessible preventive interventions. However, widely implemented interventions for adolescents that focus on opioid misuse prevention with mental health components are lacking [16]. As over 90% of adolescents are enrolled in schools and prior school-based interventions have demonstrated feasibility for substance misuse prevention [17,18], educational institutions offer a prime setting to meet adolescents where they are [19,20]. However, existing preventive interventions directed at opioid misuse among adolescents and young adults have either been conducted outside of the school setting [21,22], such as the emergency department [22], or require the involvement of trained clinicians [23-27], both of which can limit wide scalability.

Digital interventions, such as serious video games, have shown promise for targeting health outcomes among adolescents and young adults [17,18,28-30]. Given the near ubiquity of mobile device ownership in this population, over 90% of adolescents and young adults own mobile devices [31]. Digital interventions are also widely scalable and can reach more adolescents than would be possible with in-person interventions, particularly in light of the limited availability of trained personnel [32]. Serious video games—video games designed for a primary purpose other than entertainment [17,18,28-30,33]—are effective as interventions for adolescent substance misuse [34] and are a popular medium for adolescents, as over 95% of adolescents play video games [35]. Through simulated role-playing, which is a highly effective method used for learning purposes [36], serious video games allow individuals to practice behavioral skills in a safe and entertaining manner [37,38], increasing the likelihood of translating these practiced skills into real-life situations [39,40]. Because the same content is delivered to all adolescents in the same format, video games can allow for fidelity of the intervention content [41], allow for precise monitoring of how much content each adolescent has accessed [42], and may be more cost-effective [43]. Video game interventions have demonstrated efficacy in affecting behaviors related to health promotion and disease management in many areas, including chronic diseases, healthy lifestyles, and mental well-being [17,29,30,44-50]. Existing video game interventions that target opioid misuse among adolescents are limited [51,52], have not focused specifically on older adolescents [52], are not widely implemented, have yet to be rigorously evaluated in the school setting, and do not specifically address mental health risks [51,52].

In this paper, we describe the formative work of developing a serious video game intervention, *PlaySmart*, to prevent the initiation of opioid misuse among 16- to 19-year-olds at a higher risk for substance misuse. These efforts build on previous work at the play2PREVENT Lab, which developed and evaluated evidence-based video games for prevention among children, adolescents, and young adults [17,18,29,53,54]. *PlaySmart* is funded through the National Institute of Health Helping to End Addiction Long-Term Prevention Cooperative (HPC) Initiative. The National Institute on Drug Abuse ’s primary call for HPC was to address the opioid crisis among those who experienced greater risk among youth and young adults aged 16 to 30 years. HPC consists of a coordinating center and 10 research projects funded to test and implement evidence-based interventions designed to prevent the onset or escalation of opioid misuse among at-risk groups of youth and young adults.

### Aims

The aims of the formative research were to (1) conduct focus groups and interviews to inform the content and storylines of the video game, *PlaySmart,* designed to prevent the initiation of opioid misuse among adolescents aged 16-19 years; (2) develop and conduct a preliminary evaluation (playtesting) of a video game, *PlaySmart*, in partnership with Schell Games and the National School-Based Health Alliance (SBHA) to be evaluated in a subsequent large-scale randomized controlled trial (RCT); and (3) conduct focus groups and interviews among key stakeholders to understand facilitators and barriers to implementing *PlaySmart* in school settings. An RCT is currently underway to evaluate the efficacy of this video game.

## Methods

### Theoretical Frameworks

A total of 3 phases of formative research (development, playtesting, and preimplementation) were guided by specific evidence-based theoretical frameworks.

#### Frameworks That Informed Development of Game Content

*PlaySmart* development was informed by 3 established theories: the health belief model [55], the theory of planned behavior, and social cognitive theory [56]. These theories describe how various ecological (individual, interpersonal, and environmental) factors influence the risk of engaging in substance misuse. The health belief model suggests that the likelihood that an individual will partake in a particular behavior (opioid misuse) is driven by their belief in how susceptible they are to its negative consequences and how effective they think a behavior will diminish their susceptibility to these impacts [55,57]. The health belief model consists of 6 constructs: perceived susceptibility, perceived severity, perceived benefits, perceived barriers, cues to action, and self-efficacy [57,58]. The theory of planned behavior posits that an individual’s behavior (opioid misuse) is driven by intentions that are influenced by attitudes, subjective norms, and perceived behavioral control (self-efficacy, external factors, and resources) [59-61]. Attitudes toward drug use and intentions to misuse are strongly influenced by the perception of the risk of harm [62]. As such, effective prevention interventions target these antecedent factors [28,30] to prevent eventual misuse. Social cognitive theory posits that learning occurs within a social context through an individual’s interaction with their environment [63] and that the likelihood that an individual will engage in a behavior (opioid misuse) is driven by the individual’s past experiences (reinforcement) and how the individual is influenced by internal factors (capability, self-efficacy) and their external environment (expectations, modeling) [63]. Social cognitive theory consists of 6 constructs: reciprocal determinism, behavioral capability, observational learning, reinforcement, expectations, and self-efficacy [63]. Improving self-efficacy is informed by the health belief model, the theory of planned action, and social cognitive theory and has been used in other video game interventions [28,30,34,64,65] targeting behavior change [65,66] such as substance misuse among adolescents [34] and found to be effective [67].

*PlaySmart* video game development was grounded in these theories with focus group and interview guides informed by them, and theoretical components are reflected in the game content (see focus group guide in Multimedia Appendix 1). *PlaySmart* aims to increase the perceived risk of harm from opioid misuse (primary outcome), as it has been shown to be inversely related to actual drug use [16,68,69]. Prior studies have demonstrated that older adolescents with low perception of the risk of harm from opioids are significantly more likely to misuse opioids [68,70]. The game provides opportunities to practice skills such as refusing misuse of opioids that bolster self-efficacy, model adaptive behaviors by observing the actions of other nonplayer characters in the game, and highlight avenues for resources that the player can relate to in their own life.

#### Framework That Informed Approach to Game Development

To guide our approach to game development, we used user-centered design principles. User-centered design is an iterative process that prioritizes the end user throughout the design and development phases [71,72]. It begins by identifying the needs of the end user and recognizing that their goals will ultimately determine the effectiveness and usefulness of the game. This is followed by the actual development of the game and its evaluation. A user-centered approach typically includes the use of multiple modalities, including focus groups, interviews, surveys, and user testing. Evidence has demonstrated that user-centered design increases the likelihood that interventions will be used by the end user [64,71,72] and has been used in the development of other digital interventions. A user-centered design has been used in the development of other digital interventions for substance use prevention among adults [73,74] and adolescents [75].

#### Framework That Informed Preimplementation

To inform the future implementation of *PlaySmart* in school settings, we conducted preimplementation focus groups to assist in understanding potential anticipated facilitators and barriers that could affect future implementation of *PlaySmart* in school-based settings. Preimplementation activities were informed by the Exploration, Preparation, Implementation, and Sustainment (EPIS) framework [76]. The EPIS framework is an evidence-based tool that guides and facilitates its successful implementation. During the Exploration phase, the needs of the target end user are identified to determine the best practices that would ensure that those needs are met by the new product [76]. The Preparation phase allows potential barriers and facilitators to be successfully implemented and a plan for implementation to be developed [76]. During the Implementation phase, the developed plan is conducted with ongoing monitoring [76]. During the Sustainability phase, progress is monitored on an ongoing basis to identify when adaptation might be needed [76]. The EPIS framework has been successfully used in implementation programs for youth prevention [77]. During this formative work, we conducted activities in the Exploration and Preparation phases.

### Design

Formative work for the *PlaySmart* video game intervention consisted of 3 phases spanning 2 years, from early 2020 to late 2021 (Table 1). Phase 1 consisted of focus groups and interviews with a variety of stakeholders to inform the development of video game intervention. During phase 2, pilot playtesting and focus groups with adolescents were conducted to gather feedback on game content and experience, which informed the finalization of the video game. Phase 3 consisted of focus groups with school-based providers to better understand the factors to consider for successful future implementation of *PlaySmart* (preimplementation).

**Table 1:** Overview of aims and participant activities by phase.

Phase, primary aims, and group description				Number of focus groups or interviews		Participants (N=166), n (%)
**Phase 1 (2020)**						
	**Develop content for *PlaySmart* video game**					
		Youth from 4 high schools in Connecticut	7 focus groups		37 (22.3)	
		SBHA^a^-established YAC^b^	3 focus groups		15 (9)	
		SBHA personnel	5 focus groups		25 (15.1)	
		OUD^c^ treatment providers	6 interviews		6 (3.6)	
		Individual in treatment for OUD	1 interview		1 (0.6)	
		FCD^d^ prevention specialists	1 focus group		6 (3.6)	
**Phase 2 (2020-2021)**						
	**Playtesting for *PlaySmart***					
		High school aged youth who played through all or portions of the game	9 focus groups		33 (19.9)	
**Phase 3 (2020-2021)**						
	**Explore and identify potential barriers and facilitators to implementation**					
		SBHA personnel	6 focus groups		26 (15.7)	
		SBHA-established YAC	3 focus groups		17 (10.2)	

^a^SBHA: School-Based Health Alliance.

^b^YAC: youth advisory council.

^c^OUD: opioid use disorder.

^d^FCD: Freedom from Chemical Dependency.

### Participants

#### Development

Adolescents and adults from multiple local and national stakeholder organizations engaged in the development of *PlaySmart* through focus groups and individual interviews. Adolescents were recruited from our local partner high schools and targeted e-mails to the SBHA and their youth advisory council (YAC) members. SBHA is a nationwide organization that advocates school-based health care for children and adolescents through the support of school-based health centers across the country. The SBHA oversees over 2500 school-based health centers in 49 states and Washington, DC. The YAC is a youth service group established by the SBHA and is composed of adolescents aged 16 years or older who are passionate about advocacy and share their expertise on health topics that affect students’ age. Adults recruited for the focus groups and individual interviews consisted of adult affiliates of SBHA, OUD treatment providers, an individual in treatment for OUD, and prevention specialists. SBHA adult affiliates were recruited through announcements, and flyers sent through internal listserves. OUD treatment providers and individuals were recruited from the APT foundation, a nonprofit organization and one of the oldest treatment centers in the United States. Finally, prevention specialists were recruited with assistance from officials at Freedom from Chemical Dependency Prevention Works, an international nonprofit organization that aims to provide substance misuse prevention programs in schools. All participants from the development focus groups and interviews received one US $30 gift card for their time.

#### Playtesting

Participants from the playtesting focus groups were recruited using informational flyers and social media advertising on Facebook and Instagram. Owing to COVID-19, the research team had to adapt to web-based recruitment methods and focus group procedures. Web-based recruitment methods involved distributing electronic posters to different social media platforms and via listservs of partner organizations, such as SBHA. Participants either participated in (1) a 2-hour session that consisted of playing a portion of the game, answering follow-up qualitative questions, and receiving US $30 for their participation or (2) six 1-hour sessions that consisted of playing the entire game, completing procedure-based follow-up questions, and receiving a total of US $60 for their participation.

#### Preimplementation

SBHA adult affiliates and YAC members were recruited during 2 of the annual national SBHA conventions. Participants in these groups received a US $40 gift card in 1 focus group session.

### Procedures

#### Development

The focus groups and interviews ranged from 60-90 minutes in duration. Questions focused on knowledge of opioids, perception of the risk of opioid misuse, factors contributing to misuse, and narratives from those who were opioid naive or who had personal experience with opioids, such as providers, prevention specialists, or individuals in treatment (Multimedia Appendix 1). In addition, focus groups asked about current game use and what games (if any) participants engaged with to gauge their familiarity with playing games and get a sense of the prevalence of gamers in the focus groups. Focus groups and interviews were audiotaped. Two investigators attended each focus group and conducted the interviews. One investigator led the discussion (facilitator), whereas the other took notes on observable behaviors (note-takers). After each focus group, the investigators compiled a debrief summary. Audiotapes from the focus group and the interviews were transcribed using third-party services.

#### Playtesting

To assess and improve the acceptability and usability of *PlaySmart,* we conducted 60- to 90-minute playtesting sessions with adolescents. Each playtesting session consisted of 2 sessions. During the first part, the participants played the game. During the second part, they completed assessment questions using either surveys (1 group) or debriefing focus groups (7 groups). See Multimedia Appendix 2 for the questions asked during playtesting.

One group played through the entire game and completed 3 surveys on knowledge, perception of risk of harm, and gameplay experience. The knowledge survey was developed by the team consisted of 5 true or false questions related to knowledge about opioids (eg, “prescription opioids can be just as deadly as heroin when they are misused”). The perception of risk survey consisted of 5 questions from the Monitoring the Future Study related to participants’ perception of the risk of harming themselves from different forms of opioid misuse (eg, “How much do you think people risk harming themselves (physically or in other ways), if they try heroin once or twice?”) [78]. Options for the risk of harm scale consisted of no risk, slight risk, moderate risk, and high risk. The gameplay experience was assessed using 8 statements on how participants felt playing the video game. Sample questions include “Playing *PlaySmart* was interesting.” Responses ranged from 1 (not at all) to 5 (a lot).

The remaining 7 groups played different aspects of the video game (storylines and minigames) and responded to open-ended questions. We asked the participants what they thought the purpose of the game was, what they learned from playing the game, what challenges they experienced while paying the game, if gameplay prompts were easy to understand, if the storylines were realistic, and recommendations to improve the game.

#### Preimplementation

To optimize the implementation of *PlaySmart* in school-based settings*,* a total of 9 focus groups with adults from SBHA personnel and adolescents from the SBHA YAC were conducted. These groups focused on facilitators and barriers, as well as how school-based health officials might be able to implement the game, possible motivations for adolescents to engage with the game, and how the game might be used in their communities. The focus groups were 60-90 minutes in length. Two investigators attended each focus group. One investigator served as the facilitator, whereas the other investigator took notes. Following the completion of each focus group, the team met for a debriefing session and a summary from the debriefing session was compiled.

### Analysis

#### Development

Our analysis was guided via the thematic analysis by Braun and Clarke [79] to provide storylines, character depictions, and other patterns noted in the data. After each focus group and interview, debriefing summaries and notes were completed by the note-taker and reviewed by the facilitator to generate salient themes. Then, using a reflexive approach [79], conceptualized patterns from the transcriptions were added. The research team iteratively reviewed and updated the content until 6 distinct storylines emerged.

#### Playtesting

Data from the participant feedback were qualitatively analyzed to identify common themes. Analyses focused on players’ gameplay experience, and input was used iteratively to improve the game content and design. In this phase, we also assessed knowledge through an analysis of participant feedback on gameplay mechanics and character relations. With the game nearing its’ final form, we verified through participant responses that they understood the themes of the game and acquired the knowledge we were targeting. Because of the limited number of participants (n=3) who completed the quantitative survey on knowledge of opioids, perception of risk of harm from opioids, and gameplay experience, we were unable to analyze these results (see *Limitations*).

#### Preimplementation

To analyze data from the focus groups, techniques from rapid analysis [80] were performed using notes from the focus groups and debrief summaries aligned with a template of the EPIS framework. Notes and debrief summaries were analyzed using the EPIS framework to identify salient themes on potential barriers and facilitators that could impact the future implementation of *PlaySmart*.

### Ethical Considerations

All the study procedures were approved by the Yale Institutional Review Board (#2000026247). Individuals who expressed interest in participating in the development or preimplementation phase provided institutional review board–approved informational sheets for the study. Individuals provided verbal assent if they were younger than 18 years of age and verbal consent if they were older than 18 years before participating. A waiver of parental consent was obtained for adolescents younger than 18 years. For participants who participated in play testing, written consent was obtained if they were 18 years or older. Written parental consent and assent were obtained from adolescents younger than 18 years.

## Results

### Participant Characteristics

#### Development

Among the high school and YAC adolescents who participated in the developmental phase focus groups (n=52), the average age was 17.1 (SD 0.87) years. The majority were female-identifying (33/52, 63%) and of non-Hispanic descent (37/50, 74%). Approximately one-third (17/52, 33%) of the adolescent participants identified as mixed or other race (Table 2). The SBHA adult affiliates and treatment providers were predominately non-Hispanic White female individuals with average ages of 40.8 (SD 12.9) and 46.4 (SD 9.8) years, respectively (Table 2).

**Table 2:** Demographic characteristics of participants in phase 1—development work.

Characteristics			Interview with individual in treatment (n=1)		Treatment provider interviews (n=6)		YAC^a^ FGs^b^ (n=15)		SBHA^c^ adult affiliates FGs (n=25)^d^		High school FGs (n=37)		Prevention specialist FG (n=6)
Age (y), mean (SD)			21 (NR^e^)		46.4 (9.8)		17.7 (1.1)		40.8 (12.9)^f^		16.9 (0.7)		44.7 (17.2)
**Sex at birth, n (%)**													
	Male	1 (100)		NC^g^		3 (20)		3 (13)		14 (38)		NC	
	Female	0 (0)		NC		12 (80)		20 (87)		21 (57)		NC	
	Intersex	0 (0)		NC		0 (0)		0 (0)		2 (5)		NC	
**Gender identity, n (%)**													
	Man	NR		2 (33)		3 (20)		3 (13)		14 (38)		1 (17)	
	Woman	NR		4 (67)		12 (80)		20 (87)		22 (59)		5 (83)	
	Transgender	NR		0 (0)		0 (0)		0 (0)		1 (3)		0 (0)	
**Ethnicity, n (%)**													
	Non-Hispanic	1 (100)		6 (100)		14 (93)		21 (91)		23 (62)		6 (100)	
**Race, n (%)**													
	Asian	0 (0)		1 (17)		5 (33)		1 (4)		5 (14)		1 (17)	
	Black	0 (0)		0 (0)		2 (13)		6 (26)		8 (22)		0 (0)	
	White	1 (100)		5 (83)		4 (27)		14 (61)		11 (30)		5 (83)	
	Mixed or other	0 (0)		0 (0)		4 (27)		2 (9)		13 (35)		0 (0)	

^a^YAC: youth advisory council.

^b^FG: focus group.

^c^SBHA: School-Based Health Alliance.

^d^n=23 (data were not collected for 2 participants).

^e^NR: not reported.

^f^n=21 (no age data were collected for any of the 4 participants).

^g^NC: not collected.

#### Playtesting and Preimplementation

Table 3 outlines the sample characteristics of participants in the focus groups and interviews for play testing (phase 2) and preimplementation (phase 3). Similarly, non-Hispanic female individuals comprised most of the participants in the 2 phases (Table 3).

**Table 3:** Demographic characteristics of participants in phase 2 (playtesting) and phase 3 (preimplementation).

Characteristics			Phase 2		Phase 3		
			Playtesting FGs^a^ (n=33)^b^		SBHA^c^ adult affiliates FGs (n=26)^d^		SBHA-established YAC^e^ FGs (n=17)
Age (y), mean (SD)			16.8 (0.8)		44.0 (10.5)		16.9 (1.5)
**Sex at birth, n (%)**							
	Male	19 (58)		3 (12)		4 (24)^f^	
	Female	14 (42)		22 (88)		12 (71)	
**Gender identity, n (%)**							
	Man	13 (39)		4 (16)		4 (24)^f^	
	Woman	18 (55)		21 (84)		11 (65)	
	Other	1 (3)		0 (0)		1 (6)	
**Ethnicity, n (%)**							
	Non-Hispanic	26 (79)		25 (100)		13 (76)	
**Race, n (%)**							
	Asian	2 (6)		1 (4)		4 (24)	
	Black	14 (42)^g^		7 (28)		8 (47)	
	White	11 (32)		15 (60)		3 (18)	
	Mixed or other	4 (13)		2 (8)		2 (12)	

^a^FG: focus group.

^b^One participant did not report gender identity or ethnicity. Two of the participants did not report their race.

^c^SBHA: School-Based Health Alliance.

^d^n=25 (data were not collected for 1 participant).

^e^YAC: youth advisory council.

^f^Data not collected for 1 participant.

^g^Data were not collected for 2 participants.

### Development: Focus Groups and Interviews

#### Overview

Multimedia Appendix 3 outlines the themes and accompanying quotes. Nine salient themes emerged from the development work with adolescents and providers, which were used to inform the content of *PlaySmart*: (1) mode of learning; (2) opioid identification; (3) perceived risk of harm: susceptibility, severity, sequences, or addiction; (4) prescription opioids; (5) opioid accessibility; (6) reasons to use; (7) mental health, (8) support systems, and (9) video game application: mechanics or game and content or storylines. The following section provides a brief description of each theme along with the associated key concepts that were included in the game to ensure that it was relevant, relatable, and accurate.

#### Mode of Learning

Generally, youth participants cited a lack of drug education, specifically pertaining to opioids. Even when they referenced their drug education, it was often limited to a specific grade level (eg, grade 9) or to a certain type of drug (eg, marijuana or alcohol; Multimedia Appendix 3). Many of their experiences highlighted sole exposure to opioids through the news, celebrities, or media, providing further evidence of the lack of formal opioid education in the youth population.

#### Opioid Identification

Across youth focus groups, many gaps in knowledge of opioids have emerged. Although most had heard the term “opioid” before, many were unable to accurately describe or categorize opioids. Adolescents reported that they recognized the names Percocet, Oxycontin, and Heroin, but did not classify them as opioids. This was especially true for heroin (Multimedia Appendix 3). A few participants incorrectly categorized other substances, such as marijuana, as opioids. Interviews with treatment providers confirmed this gap in knowledge, underscoring that adolescents only hold a vague understanding of opioids.

#### Perceived Risk of Harm

Drawing on this lack of knowledge, many youth participants perceived their susceptibility to risk from opioid misuse as low. A lack of opioid misuse prevalence in their age group was often cited as the primary reason for low susceptibility; many claimed that problems only existed in college students or older populations (Multimedia Appendix 3). When treatment providers reflected on their experiences with the youth, this false belief was further supported. Youth participants recognized the severity of harm from heroin but did not perceive prescription opioids to be as harmful. They attributed this logic to the mode of delivery of some opioids (eg, prescription pills and codeine drink) compared with heroin (eg, injection). Despite this, participants in both youth and provider focus groups generally understood the psychological, social, and physical harms of addiction (Multimedia Appendix 3).

#### Prescription Opioids

Youth participants often identified medical professionals as partly responsible for the opioid misuse crisis. Many shared stories of friends or close relatives prescribed opioids without proper information from the physician. Treatment providers confirmed that this was often how youth were introduced to opioids, either by their own prescription or by a prescription of someone they were close to (Multimedia Appendix 3). The participants shared that this initial prescription often led to misuse.

#### Opioid Accessibility

Participants in both the youth and provider focus groups shared that accessing opioids was often quite easy—in a medical setting, in their households, and around their peers. They attributed this to the normalized prescription of opioids and teens’ general curiosity regarding foreign substances (Multimedia Appendix 3). Youth participants shared that if it was available, it was more likely and more easily used for recreational purposes. Similarly, many participants spoke of “lean” or “dirty sprite” (formally known as codeine) as fairly accessible, easily ingestible, and widespread (Multimedia Appendix 3).

#### Reasons to Use

Participants in both youth focus groups and provider interviews highlighted an array of potential reasons for opioid misuse. Peer pressure has been frequently cited as a catalyst for this behavior, although not in the stereotypical context. This peer pressure was more implicit and geared toward blending in rather than being perceived as “cool” (Multimedia Appendix 3). The youth and provider participants also cited maladaptive coping as a reason for opioid misuse. Participants reported a variety of reasons for the need to cope, including the death of a loved one, major stress, mental health issues, and “a way out” (Multimedia Appendix 3).

#### Mental Health

Youth participants mentioned an abundance of mental health concerns; some were related to opioid misuse, whereas others were independent of it. They specified a wide range of stressors that contribute to mental health issues and opioid misuse (Multimedia Appendix 3). While recognizing these stressors, they also described their own perceptions of someone experiencing mental health issues. Youth had a wide range of opinions on seeking help. Some were supportive of professional support, whereas others expressed strong hesitance. Stigma and the lack of trust were common reasons for this resistance, so the importance of finding a good match with a provider was heavily emphasized.

#### Support Systems

Most youth participants stated that adults were a necessary support system for resilience, recovery, and well-being, although they did not agree that adults should be parents or guardians. Many shared personal advice on how to support a peer but also recognized the limitations of peer-to-peer support (Multimedia Appendix 3). Youth participants also highlighted the importance of being emotionally in tune with themselves and how that alone could serve as a form of support (Multimedia Appendix 3).

#### Video Game Application

Of the 52 youths, 41 (79%) who participated in the development work mentioned playing or having played more than one game; 11 (21%) participants did not mention playing any games. More specifically to our intervention, youth participants provided their feedback on video game details, such as mechanics, esthetics, and storyline content. Generally, they recommended that the game be simple but challenging (Multimedia Appendix 3). In terms of storylines, all youth participants provided valuable information specific to interactions with parents, situations at parties, and even route friendships could take.

### Development: PlaySmart Video Game Intervention

On the basis of the information gleaned from our development focus groups and interviews, our research team collaborated with our game development partners, Schell Games, to launch and finalize the video game intervention, *PlaySmart.* Game content was directly informed by focus groups and interviews, similar to other digital interventions targeting adolescents [29,30,53,54,75,81-84].

The cornerstones of the *PlaySmart* video game intervention are (1) six storylines that form the narrative foundation: *Trading Wisdom*, *Lean on Me*, *A Friend in Need*, *Grandma’s Pills*, *Tough Love*, and *A New Direction*; (2) six minigames that provide skill-building opportunities: *Risk Sense*, *Stress Sense*, *Refusal Power*, *Social Media Game*, *Choice Power*, and *Know Power*; and (3) a participant tracker: *Future Sense*. At the beginning of the game, players review their future self in *Future Sense* to see how poorly their lives turned out because of opioid misuse and are tasked with changing their future by playing *PlaySmart*. The use of the future self-motivates players to do better in the present by drawing on evidence that shows that imagining the future self and highlighting similarities between the current and future self can influence positive behavior change [85].

These 6 storylines encompass major topics that address opioid misuse and mental health. In *Trading Wisdom*, the player learns to use opioid prescriptions safely, the consequences associated with misuse, and nonpharmacologic and nonopioid alternatives for pain management. In *Lean on Me*, the player learns about high-risk situations where opioid misuse is likely to occur (party), the role of peer pressure, how to refuse an offer to use opioids, and the dangers of misusing opioids. In a *Friend in Need*, the player learns about how mental stress increases the risk of opioid misuse, the benefit of seeking support from trusted adults, and how they can be supportive to their friends. In *Grandma’s Pills*, players learn about the dangers of using prescriptions that do not belong to them, including overdose and strategies from refusing opioids when offered by family members. In *Tough Love*, the player learns to set boundaries in close relationships where opioid misuse is an issue, and how to support people close to you who are struggling with opioid misuse. In *A New Direction*, the player learns of mental health resources, existing myths around mental health and stigma and is empowered to seek mental health care when needed. Throughout gameplay, players have options to make a bad or good choice, thus practicing decision-making skills. In addition, we expect to increase the perception of the risk of harm among players through scenarios that highlight the dangers of opioid misuse. Links to other trusted websites such as the National Institute on Drug Abuse are embedded in the game and provide players with an opportunity to continue learning outside the game if they are interested.

**Figure 1:**
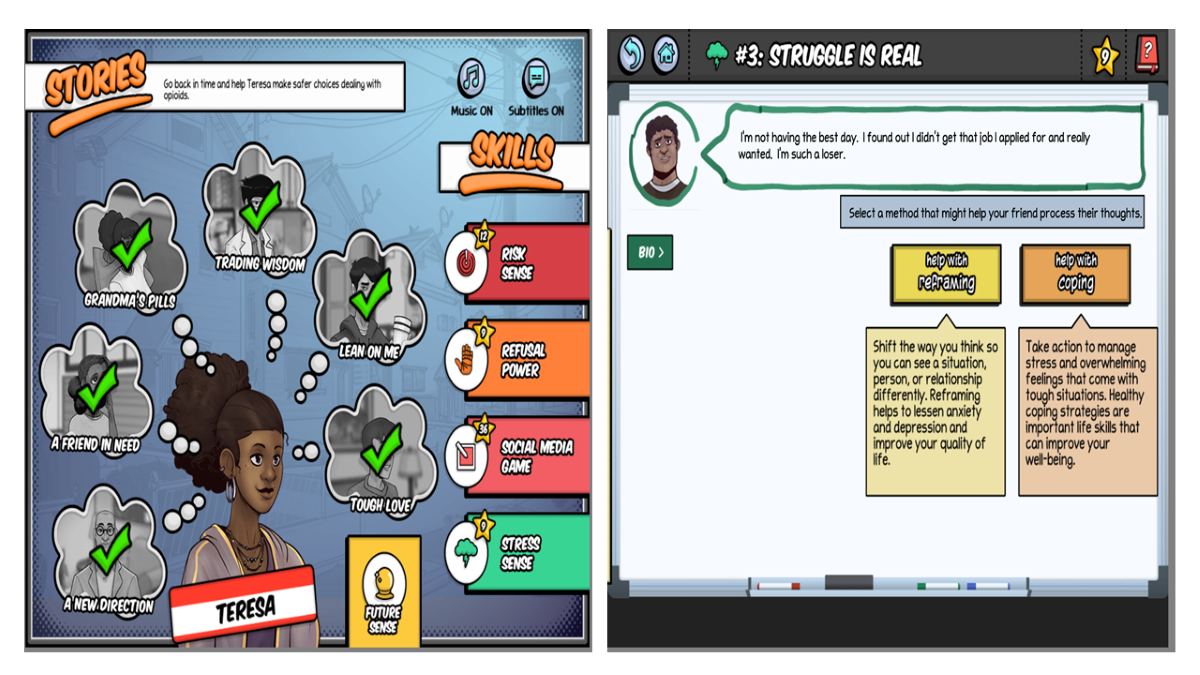
The home page of the *PlaySmart* video game on the left and a screen from the *Stress Sense* minigame on the right. Licensed under Creative Commons 4.0.

Minigames provide a real-time opportunity to improve self-efficacy through adaptive skills such as decision-making and refusal skills that are relevant for avoiding opioid misuse. In *Risk Sense*, the player is tasked with working to prevent risks (impulsivity, ignorance, and relationship problems). By playing *Risk Sense*, players become aware of the risk factors for opioid misuse. In *Stress Sense*, players have an opportunity to practice cognitive reframing, a cognitive behavioral strategy [86] to address thought distortions (all or none, catastrophizing, and should statements) and seeing a particular problem in a different light that serves to reduce the emotional distress that can increase the risk of opioid misuse [86]. In addition to cognitive reframing, players learn several coping strategies, including exercise, talking to trusted adults, watching movies and music, reading, taking time off to rest and relax, sleeping well, and using distraction techniques to take the mind of stressful thoughts. In *Refusal Power*, the player helps the character resist peer pressure to engage in risky behaviors. In a *Social Media Game*, through posts on social media, players practice advocating for behaviors that deter drug misuse, encourage help seeking from trusted adults and mental health professionals, and support peers who are struggling. In *Choice Power*, players learn to communicate effectively with others to get the help they need or to avoid getting into trouble. In *Know Power*, players learn facts about opioids and opioid misuse through conversations with other nonplayer characters. *Choice Power* and *Know Power* are embedded within each storyline, whereas the other minigames are shown on the homepage (Figure 1).

The final version of *PlaySmart* is a web-based video game that is accessible to any device that can access the internet and has a browser. User data were stored in an SQLite database hosted on the Amazon Web Services. Data on players’ progress in the game, such as what game levels have been completed and how many minutes were spent on the game, can be downloaded securely from the database.

### Playtesting

Informed by user-centered design principles, the feedback obtained during playtesting was critical in modifying *PlaySmart* for its final form. Following the initial iteration of the game, participants were recruited to play the game. Participants generally agreed that the game was straightforward, engaging, and provided a wealth of information on opioid use and misuse. Feedback from these groups primarily consisted of recommendations for technical changes related to minigames, content of the text, or gameplay mechanics. For example, some of the feedback identified content in the game that was not relatable to adolescents and provided specific suggestions for how to improve it. Some felt that the initial set of avatars did not include enough variation to reflect a diverse group of adolescents. The use of avatars that players can relate to is critical because evidence indicates that players exhibit a higher sense of responsibility and attachment to avatars that look like them [87] and are more engaged when using avatars that look like them [88]. Some felt that some minigames needed a tutorial to orient the player. Some adolescents felt distracted by the background music and suggested a way for adolescents to modify the sound based on personal preference. Some players felt that some parts of the risk-sense minigame were not demanding, resulting in the addition of more tasks in the minigame that increased the challenge posed in the game. Other players felt that nonplayer characters in the game were too knowledgeable in contrast to their friends in real life. This feedback informed the modification of the game such that chats more realistically depicted the balance between friends who may be informed and others who may not be informed about facts relating to opioids. We used this feedback to improve the final version of the game alongside the game developers [89] (Figure 1). Through this testing, we solidified our procedures for the RCT.

### Preimplementation

Participants from our preimplementation focus groups shared the potential barriers and facilitators to implementation as well as information specific to motivation around gameplay and engagement with the intervention. SBHA adult affiliates mentioned the importance of administrative buy-in before implementing *PlaySmart* in schools and clinical settings. They also provided examples of possible ways to engage youth with the game, such as through academic incentives, making it an activity in and of itself in schools, or implementing it as part of the process of visiting a clinic (eg, in the waiting room of a clinical setting). Youth from the preimplementation focus groups generally agreed that it could be used in their school community as an interactive way to learn, and most of their first impressions highlighted several positives of the *PlaySmart* game. For example, they spoke about liking the comic-book style of the game, the relatability of each storyline, and the interactive components of the storylines and minigames. They highlighted the importance of training both staff and students to equip them with the necessary troubleshooting skills and to ensure that they understand their rights to confidentiality and data privacy. Much of the discussion regarded optimal strategies for game delivery, and many participants believed that integrating *PlaySmart* into existing structures such as health classes, clinics, or school clubs would gain the best traction from both students and staff. Focus group participants emphasized the value of incentives for implementing the game in schools, especially because it was a new program and required some human resources to implement effectively. Common suggestions for greater incentives include promoting *PlaySmart* through social media youth ambassadors, incorporating gamification elements (eg, leaderboards or communication channels), and framing it as a novel health behavior tool rather than a more traditional form of prevention-based education. This information was used to create *PlaySmarts* educator’s manual, which was shared with implementation partners as a resource.

## Discussion

### Background

The public health impact of concurrent opioid and mental health crises cannot be overstated and underscores the need for innovative approaches for engaging adolescents and young adults in preventive interventions. Given these crises, and with support from the National Institutes of Health HPC Initiative, we developed a novel video game intervention to prevent opioid misuse among older adolescents. This is the first video game intervention to address opioid misuse and mental health issues among older adolescents. Existing game-based interventions for opioid misuse have focused on younger adolescents [52] and on addressing knowledge gaps [51,52], increasing the perception of risk of harm from opioid misuse [51], improving decision-making [52], changing attitudes [51] and beliefs, and improving self-efficacy [51]. We highlight the need to address mental health as well as the significant role that underlying mental disorders play in increasing the risk for opioid misuse, address stigma, confront existing myths associated with mental health problems, and underscore the importance of addressing seeking treatment for mental disorders in addition to substance misuse.

Our formative work revealed that mental health and its connection to opioid use were among the most prevalent themes that arose from the youth focus groups. Preexisting mental health problems such as depression can increase the likelihood of substance misuse, including opioids [7-9]. In addition, the stigma surrounding access to mental health services can be a barrier to early engagement in treatment. Serious video games, such as *PlaySmart*, offer an engaging way to prioritize mental health as a central focal point by empowering adolescents to be vocal and intentional about asking for help and by breaking down myths that mental health support is ineffective or worsens symptoms. In *PlaySmart*, the risk of opioid misuse driven by underlying mental health problems is woven throughout the game, while normalizing help seeking and adaptive coping strategies. To examine the impact of incorporating mental health factors into *PlaySmart*, our ongoing RCT will also assess the impact of *PlaySmart* on mental health outcomes.

In contrast to other game-based interventions to prevent opioid misuse [51,52], and consistent with our standard practice, we engaged our target audience of adolescents as cocreators in the process, and they were involved throughout the development and playtesting of *PlaySmart*. Involving the end user increases the likelihood of relatable storylines and provides an opportunity to improve the game based on player feedback [72,73]. Most of the youth who partook in the focus groups had not previously had education focused on opioid misuse, highlighting an existing gap in the current school curriculum while also demonstrating the need for this game to adequately address. In addition, important avenues through which adolescents learn incomplete information about drugs, such as music, media, or celebrities, are directly addressed in the game.

Our youth focus groups had good racial representation, including youth identifying as Asian, Black, White, and mixed race, in contrast to a similar study focused on opioid misuse prevention among adolescents [81]. However, representation from adult representatives was predominantly White, which mirrors the racial ethnic distribution of teachers [90,91] and health care workers [92] in the United States. Further evaluation following the completion of the RCT will explore whether there were any differences in the efficacy of response among different racial and ethnic groups.

Creating evidence-based interventions without a plan for real-world implementation can negatively impact effectiveness [93]. Our preimplementation work revealed ways to facilitate the uptake of *PlaySmart* in school settings, such as incorporating the video game into existing structures, such as a health class, which would allow for greater feasibility. Other suggestions for wide-scale uptake identified from preimplementation focus groups include making *PlaySmart* available through social media or as an intervention for adolescents waiting at an outpatient clinic, particularly if these measures are incorporated into existing clinic workflows.

Video games are very engaging and universally used by adolescents and young adults. Consequently, they offer an innovative way to deliver evidence-based prevention interventions that can potentially be lifesaving. In addition, the *PlaySmart* video game is a mobile web-based intervention that can be accessed repeatedly by a player through their phones, tablets, or computers, and can be delivered anywhere with internet access. Through this formative work, we demonstrated the feasibility of engaging a comprehensive group of stakeholders (eg, adolescents with and without opioid misuse, prevention scientists, addiction medicine providers, and school representatives) in developing a compelling video game and using user-centered design principles [71,72] in both the development and initial testing of the intervention. Informed by our focus group and interview participants and guided by user-centered design principles, we translated behavior change theories, specifically the health belief model, theory of planned behavior, and social cognitive theory, into relatable storylines and minigames. Through these game components, adolescents acquire accurate knowledge and perceptions of the risk of harm from opioid misuse, learn about the benefits of addressing mental health issues, and practice a range of critical skills around refusing invitations to misuse drugs, practicing cognitive reframing when stressed, and using coping strategies and mental health resources.

### Limitations

During this formative work, specifically during playtesting, we had to switch from in-person procedures in schools to web-based procedures because of the COVID-19 pandemic. This occurred during playtesting and affected recruitment and retention during the playtesting sessions. As a result, we had only one group of participants (n=3) who played the entire game and were able to complete *quantitative* assessments of knowledge of opioids, perception of risk of harm, and gameplay experience. As such, we were unable to provide *quantitative* summary data on whether there was improvement in knowledge about opioids or perception of risk of harm from opioids in this formative evaluation. In the ongoing RCT, we are evaluating improvements in opioid knowledge and perception of the risk of harm from opioids (primary outcome) and will provide these results following the completion of the RCT. Only 1 participant with OUD participated in this study, which may have impacted the video game content, although this is a preventive intervention primarily targeting adolescents without opioid misuse. Although we assessed factors that would impact usability and engagement during gameplay, the questions did not specifically assess whether adolescents would be motivated to play the game. We are currently assessing factors that would influence motivation in ongoing RCT. Future formative work can assess the factors that can influence motivation, which may influence the final game design. We assessed how familiar adolescents were with video games during the focus groups, but we did not assess perceptions of the game between those who were hard-core gamers and those who were not, which may have informed game design to have the broadest reach across different levels of gamers. Future formative studies on game development should include this information.

### Conclusions

The ongoing dual opioid and mental health epidemics underscore the need for effective prevention interventions that can engage adolescents and address accurate information and skill-building in these areas. Video games provide an appealing medium to engage adolescents while translating evidence-based theories into a digestible, easily accessible format that allows for repeated practice of behaviors necessary to prevent opioid misuse and promote mental health in adolescents in real-world settings. Engaging the target audience and other key stakeholders in the development and testing of this digital health intervention ensures that it reflects their voices and perspectives for the greatest impact and reach.

## Appendix

Multimedia Appendix 1 Focus group guides.

Multimedia Appendix 2 Playtesting assessment questions.

Multimedia Appendix 3 Themes and sample quotes from PlaySmart development.

## Abbreviations

EPIS: Exploration, Preparation, Implementation, and Sustainment
HPC: Helping to End Addiction Long-Term Prevention Cooperative
OUD: opioid use disorder
RCT: randomized controlled trial
SBHA: School-Based Health Alliance
YAC: youth advisory council

## Data Availability

We have provided our focus group guide, synthesized results from the focus group analysis, and questions used during the playtest. Owing to the sensitive nature of the data from the focus groups, including the presence of potentially identifiable information, we cannot share the data from the focus groups.
