# Supplementary material for: A Video Game Intervention to Prevent Opioid Misuse Among Older Adolescents: Development and Preimplementation Study
Source: JMIR Serious Games. 2023 Nov 3;11:e46912. doi: 10.2196/46912 (PMC10656656; doi:10.2196/46912)
Supplement: Multimedia Appendix 1 [file games_v11i1e46912_app1.docx]

**HEAL: Focus Group Guide**

**Protocol: 2000026247**

Warm up: (2 minutes):

1. **What videogames are you playing right now? What do you like about it/them?**
2. **Have you ever learned something from playing a videogame?**
   1. Did you remember what you learned? What made you remember it?

Substance Use—General (5 minutes):

1. **What kind of substances (e.g., tobacco products, marijuana, alcohol, etc.) do you think teens your age experiment with?**
   1. Possible prompt: Why do you think teens your age experiment with them?

Opioids (45 minutes): (5 minutes initial thoughts, 20 minutes perception of risk of harm, 20 minutes sharing experiences)

As you know, our focus in today’s discussion is on opioids.

Opioids are a group of substances that can be broken down into two categories: the first is medications that can be prescribed for pain and the second is illegal drugs like heroin. Some examples of prescription opioids include Percocet, Vicodin, oxycodone, codeine and morphine. Fentanyl is the name of another prescribed opioid that is particularly strong and can be combined with heroin, making it very dangerous.

1. **What are some of your initial thoughts after hearing me read that?**
2. **How many of you have heard of the term opioids?**
   1. If yes:
      1. What have you heard about them? What are some examples of opioids that you have heard of?
      2. How have you heard about them?
         1. Prompt: media, music, friends, family, etc
   2. If no, what did you think opioids are?
   3. Have you heard of these terms: O.C., Oxy, Percs, Vike, M, Monkey, White stuff, Lean, Schoolboy, Sizzurp, Purple Drank, Loads? Tell me what you know about these terms, if anything at all.

Perception of Risk of Harm (as adapted from the Health Belief Model) (20 minutes): [Hypothetical Questions]

Now I’d like to ask you some questions about opioid *misuse*. As I explained earlier, prescription opioid misuse refers to using prescription opioids in any way other than prescribed, for example, using someone else’s prescription or taking opioids to get “high.”

1. **What are some consequences for misusing opioids?**
2. **Do teens your age believe opioids are dangerous? Why or why not?**
3. What about prescribed drugs like Percocet, Vicodin, codeine, or oxycodone?
4. What about illegal drugs like heroin?
   - 1. Possible Prompts: [Depending on response.] Why do you think teens believe X is more dangerous than Y?
     2. Can you share a story about a time that led you to believe that X was more dangerous than Y?
5. **Why might teens misuse opioids?**
6. **What do people your age think about other teens who misuse opioids?**
   1. Possible prompts: Describe what you think this teen would look like, act like, and give a scenario for this teen - Where are they? Who are they hanging out with?

Stories/Experiences (20 minutes)

Thank you for sharing your thoughts. Now I’d like to ask you to share some stories.

1. **Can you think of a time when someone you or someone you know was prescribed an opioid?**
2. Possible prompt: For example, for sports injuries or surgeries like removing wisdom teeth?
3. **Can you think of a time when someone you know misused an opioid? (ie after being prescribed, or for recreational use)**
4. Possible prompt: What happened? Who was there? Where did it happen?
5. Possible prompts: How did they handle the situation? What made them say yes to the offer or refuse the offer?
6. **What advice would you give someone in how to handle that kind of situation in the future?**

Now, I would like to talk about the addictive nature of opioids.

Addiction: (10 minutes):

1. **When I say the word addiction, what comes to mind? Let's go around the group.**

Thank you for all of this information. Prescription opioids can be highly addictive because they can make someone feel very good or “high,” which can lead individuals to misuse them. Side effects of opioid addiction include becoming physically dependent (meaning you need to take them to feel normal), and there is a risk of overdose. Overdose means you become unconscious and can stop breathing and die.

Addiction is a complex condition where individuals have an uncontrollable physical urge to use a substance(s), despite harmful consequences. People with addiction have an intense focus on using a certain substance(s), such as alcohol or drugs, to the point that it takes over their life. They keep using alcohol or a drug even when they know it will cause problems.

1. **How does someone become addicted to opioids?**
   1. **Possible prompt: Talk me through the timeline from first use to addiction. (ie if someone was prescribed it, tried it at a party…what happens from there?)**
2. **What might make teens *more* at risk of potentially developing an addiction to opioids?**
3. What is their life like?
4. **Imagine a friend was struggling with an opioid addiction.**
   1. What would they look like? (physical characteristics and symptoms)
   2. How would they be acting? (ie, out of character)
   3. How would you help them handle their situation?
   4. Who could they go to for help? (family, teacher, counselor, etc.)

As you know, our goal is to build a videogame that prevents kids from experimenting with opioids in the first place. I’ve asked you about your thoughts and experiences, and now I would like you to use your imagination in thinking about how this can be applied to a videogame.

Videogame Application (10 minutes):

1. **Can you think of some specific scenarios that would feel relatable to teens your age for this type of videogame?**
2. Places? Characters?
3. Scenarios?
   1. Doctor scene
   2. Party scene
   3. Finding prescription in bathroom cabinet scene
   4. Sports injury scene
   5. **Any other scene?**
4. What could happen based on the decisions made?
5. **Based on what we’ve talked about today, what do you think teens your age should know about opioids?**
6. **What kinds of skills do you think teens could practice in a videogame in order to prevent an addiction to opioids?**
   1. **Prompt: peer pressure, recognizing dangerous choice**
7. **[Show artwork.] Tell me about the characters in this picture.**
8. What do you think this person is doing?
9. Why do you think this person is considering this?
10. Do you think this person is at risk of harming herself? Tell me more.
11. **Is there anything that you would like to add or share about what we’ve discussed today?**
