# Supplementary material for: A Video Game Intervention to Prevent Opioid Misuse Among Older Adolescents: Development and Preimplementation Study
Source: JMIR Serious Games. 2023 Nov 3;11:e46912. doi: 10.2196/46912 (PMC10656656; doi:10.2196/46912)
Supplement: Multimedia Appendix 2 [file games_v11i1e46912_app2.docx]

**Playtesting Questions**

***Knowledge Questions***

1. True or False: Opioids are a group of medications or drugs that includes both prescription pain medications and illegal drugs like heroin.
2. True or False: Prescription opioids can be just as deadly as heroin when they are misused.
3. True or False: Opioids only come in pill form.
4. True or False: A person who is addicted to opioids can stop taking them whenever they want.
5. True or False: Opioids can alter judgment which can cause you to do risky things you Tyou drove while under the influence of opioids.

***Perception of Risk of Harm***

How much do you think people risk harming themselves (physically or in other ways), if they :

1. Try heroin once or twice
   1. No risk
   2. Slight risk
   3. Moderate risk
   4. Great risk
2. Try heroin once or twice without using a needle
3. No risk
4. Slight risk
5. Moderate risk
6. Great risk
7. Try any narcotic other than heroin (Non-heroin narcotics such as prescription opioids such as Codeine, Vicodin, OxyContin, Percocet, etc.) once or twice
8. No risk
9. Slight risk
10. Moderate risk
11. Great risk
12. Take any narcotic other than heroin (Non-heroin narcotics such as prescription opioids such as Codeine, Vicodin, OxyContin, Percocet, etc.) occasionally
13. No risk
14. Slight risk
15. Moderate risk
16. Great risk
17. Take any narcotic other than heroin (Non-heroin narcotics such as prescription opioids such as Codeine, Vicodin, OxyContin, Percocet, etc.) regularly
18. No risk
19. Slight risk
20. Moderate risk
21. Great risk

***Gameplay Experience***

*Rated on a scale of 1-5 (1 = not at all; 5 = a lot)*

1. This game helped me.
2. Playing *PlaySmart* was interesting.
3. I was absorbed in this experience.
4. I felt frustrated while playing *PlaySmart*.
5. I found *PlaySmart* confusing to use.
6. I liked the art and design of *PlaySmart* (meaning you liked the way it looked).
7. I understood the stories in *PlaySmart.*
8. I felt like I was in control of the game.
