# Supplementary material for: A Video Game Intervention to Prevent Opioid Misuse Among Older Adolescents: Development and Preimplementation Study
Source: JMIR Serious Games. 2023 Nov 3;11:e46912. doi: 10.2196/46912 (PMC10656656; doi:10.2196/46912)
Supplement: Multimedia Appendix 3 [file games_v11i1e46912_app3.docx]

Appendix 3.

| Theme | Subtheme | Quotes |
| --- | --- | --- |
| Mode of Learning | Educational | *Female adolescent: “I feel like yeah, they just need to start teachin’ it more. They only teach it about once in middle school, and it’s not even like how bad they are. Like they’ll just teach about weed and how weed is such a bad drug, like it’s crazy bad. But they won’t teach about all these other drugs that doctors prescribe to people, and how doctors are basically creating addicts, and creating all these problems.”* |
|  | Media/News | *Male adolescent: “Oh yeah. I’ve heard of them. I’ve seen Fentanyl in the news, like how people lace things with it. People, of course talk about it, like they don’t buy street carts* *or anything because they might be laced with something.”* |
|  | TV/Celebrity/Music | *Female adolescent: “I think that like a lot of like famous people and celebrities, they like promote it. Not intentionally, but a lot of them do it because they have to constantly stay like energized and stuff when they’re performing…”* |
| Opioid Identification | Recognition/Description | *Intersex adolescent: “I think a lot of teens might know, like they have probably heard of them at some point in their lives, but there are probably a select few that know what most of them do, or the difference between prescription opioids and illegal opioids.”*  *Female adolescent: “Well I think a lot of people know like certain drugs are obviously bad. But you learn these are drugs and these are bad but they don't really break them down into these are the categories of drugs. And what falls under each category. So a lot of people know the names but they won't know what category they're under.”* |
|  | Label/Name | *Male adolescent: “Oh, yeah, like I've heard about morphine, heroin, and I haven't heard about the other ones.”*  *Interviewer: “Okay. Any specific opioids that you see more commonly?”*  *Male adolescent: “Weed or Percocet.”* |
| Perception of Risk of Harm | Susceptibility/Relevance | *Male adolescent: “You see these kind of stories on the news all the time. There’s kinda’ like this idea that you’re the protagonist of your own story and nothing bad can happen to you.”*  *Female adolescent: “Yeah. ‘Cause I feel like college kids are mostly more so use these kind of stuff, ‘cause you know how like people say that college kids have parties and stuff like that, but I don’t really see like high school kids probably experiencing with these.”* |
|  | Severity | *Female adolescent: “I feel like heroin’s really, like that’s worse, ‘cause in my opinion I feel like heroin’s like a suicidal drug. Like yeah, dirty Sprite’s bad, but all it does is basically make your heart rate slower, you’re just laying there, and you sleep. I don’t know, I just feel like heroin’s so much worse. Like if you want to end something, you want to like kill yourself, basically that’s the drug to go, ‘cause that’s like so hard.”*  *Male adolescent: “With pills, they seem more normal. You just pop one and it’s fine. But when you think of a needle, like if you’re in a drug house or with a bunch of friends, and you’re just passing that needle on, you don’t know what someone might have. They could have something else in their bloodstream, and you’re just sharing it, and on and on. That should make anyone freak out. Never try that. But when it comes to pills, it’s like no one’s put their mouth on this, no one’s blood has been in this. It’s just a pill. I can just take it.* |
|  | Consequences/Addiction | *Female adolescent: “Well I know some certain drugs, I don't know the names, but I know you can do it once and then you can be addicted by the next day. And I feel like every day as in you have the resources to take it every day but you could also be addicted if it's presented to you. If you know there's a way you can get it every so often then I feel like if oh, I'm going to go to a party. And I know so-and-so is going to have this type of pill. You look forward to taking it. So I feel like that's in a way you're addicted because that's why you're going to go there just to”* |
| Prescription Opioids | Transition to Misuse | *Female adolescent: “So when you’re prescribed something that’s supposed to help you with the pain, you become reliant on it. So you think it’s always gonna’ help you, but you don’t really need it after a certain point, ‘cause you just think you’re still in pain ‘cause you’re used to takin’ it so much. So they don’t realize they’re addicted to it. They just think oh, it’s a part of my life now, so I have to take it to feel okay.”* |
|  | Doctor’s Role | *Female adolescent: “I feel like it’s the doctor’s job to tell you the pros and cons about the drugs that they’re giving you, and it says it on the bottles too, pros and cons of the drug that they’re giving you. And now lately, everybody’s always getting hurt. Like you go fall down the stairs and they’ll prescribe you those drugs. Like it’s not hard to get them from doctors either.”*  *Male adolescent: “One of my friends, she burned her hand while she was cooking, and her doctor prescribed her morphine for it. I never really asked her if she felt it was dangerous or whatever. She just kind of took it and she said it kind of made her feel out of it. I don’t really think her doctor talked to her much about the dangers of abusing it. The doctor kinda’ just prescribed it.”* |
| Opioid Accessibility | Process | *Male adolescent: “Everybody’s shootin’ it up with needles, and with the problem with that is, if you shoot it up in the wrong vein, it’s over for you, and like with Lean it’s easier to just get…All you do is literally just mix two liquids and you just start drinkin’”* |
|  | Acquisition and Distribution | *Female adolescent: “I feel like these are things that people can easily find in their house, like if one of their parents or themselves have gotten hurt, and they’ve been prescribed it by a doctor, it just stays there, ‘cause…Or they don’t use it at all, or they just continue to be in pain, but they’re not really in pain. So they just have it stocked up in their house, so it’s just easily accessible to them.”*  *Male adolescent: “I believe like after first use, I think that’s kind of where you determine whether or not you enjoy or not, and if you do enjoy it will become kind of like a recreational thing since you already have availability to it.”* |
| Reasons to Misuse | Risk Factors | *Male adolescent: “When people talk about peer pressure, they kinda’ talk about people directly telling you to do something, but then they kinda’ forget like you can be pressured just be people around you doing it.”* |
|  | Recreational | *Male adolescent: “I also kind of feel like there’s a curiosity aspect to it too, because I feel like people see everyone else smoking, and they kind of think like what about it makes it so enticing, so they then also smoke, and then they find out that they like it, and then they just do it whenever they’re bored.”* |
|  | Coping | *Male adolescent: “I think they do drugs and they try different drugs as a coping mechanism, because if somebody goes through a traumatic stage in their life or something, they lose a family member, and they don’t got nobody to talk to, they use…They substitute that person with the drug, and once that drug makes ‘em feel some type of way, maybe I won’t remember it no more, they’re gonna’ go back and keep doin’ that drug because they don’t remember that story or that tragedy that happened, so they stick to the drugs and keep doin’ the drugs.”*  *Female adolescent: “Yeah, like [female adolescent] said, I feel like they see it as a means of just solving your problems. There's no other option to solving problems or solve their problems. So this could be the only way out of it, that type of thing.”* |
| Mental Health | Stressors | *Female adolescent: “They could have problems with their significant other or bad grades in school or family issues.”*  *Interviewer: “What would be an example of a family issue?”*  *Female adolescent: “Maybe if their parents are getting divorced or if their sibling is going away for a while. Like if they're in the military or something like that.”* |
|  | Changes in Behavior | *Female adolescent: “I don't know if this is something, but say you wake up at 6:00 in the morning every single day. And then you start taking pills and then you realize like you wake up at 11:00 in the morning. And I don't know. Some changes from what you normally do or if you look different or if you, stuff like that.”*  *Interviewer: “Okay.”*  *Female adolescent: “Adding onto [female adolescent]. I feel like changes in your daily routines or changes physically, socially and mentally…”* |
|  | Providers | *Male adolescent: “Some of my friends who may have depression or anxiety, they, there's kind of a preconceived notion with them that talking to someone is not going to help. They tried that. They did that. And I think, I don't know how to get over that.”*  *Intersex adolescent: “I mean, that's how a lot of friends that basically, their mindset was, if they were going to therapy, they thought that they had to be fixed. And for some of them, they thought that the reason that they were was because something was wrong with them, and that no one wanted to be around them unless they would go. So, they thought that something was definitely wrong and that nobody truly cared about them because of that.”* |
| Support Systems | Parent/Guardian | *Female adolescent: “I feel like most…You would suggest to some people to go to their parents, but they don’t feel comfortable goin’ to their parents about it, ‘cause they feel like their parents won’t understand them, or listen to them, or get what they’re going through.”* |
|  | Trusted Adult/Other Adult | *Male adolescent: “Any adult figure that you have trust instilled in. It doesn’t necessarily have to be somebody specialized in the medical field. It doesn’t have to be somebody that is into social work or to helping children benefit. It’s just vocalizing it and getting it out, and somebody understanding you. Somebody that has some type of power, ‘cause although we could talk to our peers, our peers don’t really have any power in our lives in comparison to a teacher or an educator, anybody, any adult.”* |
|  | Peer | *Female adolescent: “I think communication is obviously the key in this. You have to obviously communicate your thoughts but I feel like if you're talking to someone that is misusing any kind of drug, you don't want to go straight to them and be like, ‘You need to stop.’ You don't - that's just you being… putting a force on top of that. You'd want to ease into it. With how is your life? What's up with your life? Anything wrong? And ease into why are you doing this. You don't want to be the first thing you say is stop.”*  *Female adolescent: “I think another point is you just be like emotionally intelligent. You should be able to understand how they may be feeling from their perspective and their past experiences. And not try to impose any kind of, any sort of your beliefs on them.”* |
|  | Oneself | *Male adolescent: “I agree with [female adolescent]. The biggest difficulty is finding your issue, I think.”*  *Interviewer: “So how do people find their issue? What do you think we need?”*  *Female adolescent: “You just…Like a time to yourself. For example, you spend time on your own. As a teen, I’m in my room all the time. Just sit by yourself and go through the things that you do daily, the things that you have to get done, the things that you need to do, and the things you want to accomplish, and you just see like oh, okay. ‘Cause eventually you’re gonna’ stumble up on something that you’re repeatedly thinking and going through.”* |
| Videogame Application | Mechanics/Gameplay | *Male adolescent: “And since you’re targeting teens, don’t make it a game for kids, like it’s so obvious. Like go to this door, open this. Treat them like they’re smart enough to realize what to do here. They might need to climb up there, they might need to pick up this. Don’t give them obvious…Once they’re not doing anything for two minutes, don’t point an arrow on the screen saying get this. Let ‘em figure it out, even it might be hard.”* |
|  | Content/Storylines | *Male adolescent: “Like how you communicate with this character, if you choose one answer, change the friendship or whatever in the long run…”*  *Male adolescent: “Yeah, I agree with that, because in, especially interactions with the other characters, because what if you do something that is kind of like, doesn't really rub the right way someone they trust, and they kind of distance themselves?”*  *Male adolescent: “I think not only hanging out with the bad people. There’s also good people that you can hang out with that will also introduce you to this, so I think it’s just something that you should…It shouldn’t be some identifiable negative person. It should be someone that’s been your best friend for a while.”*  *Female adolescent: “Yeah. They're having fun. They're drinking beer and stuff like that, and they get to the point that, where beer is not enough.”*  *Interviewer: “Got it.”*  *Female adolescent: “They don't feel adrenaline anymore and they want to try something else. And then they came, this stranger comes to them and tells them, ‘Here, try this. This will get you really high.’”* |
